# Scalp and Intracranial EEG in Medically Intractable Extratemporal Epilepsy with Normal MRI

**DOI:** 10.5402/2012/942849

**Published:** 2012-06-25

**Authors:** Tarek Zakaria, Katherine Noe, Elson So, Gregory D. Cascino, Nicholas Wetjen, Jamie J. Van Gompel, W. Richard Marsh, Fredric Bruce Meyer, Caterina Giannini, Robert E. Watson, Gregory A. Worrell

**Affiliations:** ^1^Mayo Systems Electrophysiology Laboratory, Division of Electroencephalography and Epilepsy, Department of Neurology, Mayo Clinic, Rochester, MN 55905, USA; ^2^Department of Neurology, Mayo Clinic, Scottsdale, AZ 85259, USA; ^3^Department of Neurosurgery, Mayo Clinic, Rochester, MN 55905, USA; ^4^Department of Pathology, Mayo Clinic, Rochester, MN 55905, USA; ^5^Department of Neuroradiology, Mayo Clinic, Rochester, MN 55905, USA

## Abstract

*Purpose*. To investigate EEG and SPECT in the surgical outcome of patients with normal MRI (nonlesional) and extratemporal lobe epilepsy. *Methods*. We retrospectively identified 41 consecutive patients with nonlesional extratemporal epilepsy who underwent epilepsy surgery between 1997 and 2007. The history, noninvasive diagnostic studies (scalp EEG, MRI, and SPECT) and intracranial EEG (iEEG) monitoring was reviewed. Scalp and iEEG ictal onset patterns were defined. The association of preoperative studies and postoperative seizure freedom was analyzed using Kaplan-Meier analysis, log-rank test, and Cox proportional hazard. *Results*. Thirty-six of 41 patients had adequate information with a minimum of 1-year followup. Favorable surgical outcome was identified in 49% of patients at 1 year, and 35% at 4-year. On scalp EEG, an ictal onset pattern consisting of focal beta-frequency discharge (>13–125 Hz) was associated with favorable surgical outcome (*P* = 0.02). Similarly, a focal fast-frequency oscillation (>13–125 Hz) on iEEG at ictal onset was associated with favorable outcome (*P* = 0.03). *Discussion*. A focal fast-frequency discharge at ictal onset identifies nonlesional MRI, extratemporal epilepsy patients likely to have a favorable outcome after resective epilepsy surgery.

## 1. Introduction 

The absence of a structural magnetic resonance imaging (MRI) abnormality, that is, nonlesional MRI, in patients with medically resistant epilepsy presents a medical and surgical challenge. Medical management of extratemporal neocortical epilepsy is often difficult because of high seizure burden and poor response to antiepileptic drugs (AEDs) [1–3]. The surgical management of nonlesional neocortical epilepsy is challenging due to the absence of a MRI lesion, rapid seizure propagation [4], and potential overlap of epileptogenic and eloquent brain regions. These factors contribute to the relatively poor surgical outcome because of the challenge identifying and completely resecting the epileptogenic zone required for seizure freedom [5–10].

The epileptogenic zone is defined as the brain region generating the patient's habitual seizures, and what must be resected for seizure freedom [11]. In clinical practice a preoperative hypothesis is developed for epileptogenic zone localization based on seizure semiology, interictal and ictal scalp electroencephalography (EEG), magnetic resonance imaging (MRI), and functional imaging positron emission tomography (PET) and single-photon emission-computed tomography (SPECT).

Surgical outcomes in patients with nonlesional MRI, extratemporal lobe epilepsy (NLET) are inferior compared to patients with nonlesional temporal lobe epilepsy (NLT). Reported favorable outcomes after resective surgery in patients with NLET range from 25% to 54% [9, 10, 12–21] compared to 48% to 65% for temporal lobectomy in NLT patients [22–25]. Multiple studies have directly investigated the difference in surgical outcome for NLET and MRI lesional extratemporal epilepsy. These studies uniformly show a significantly higher likelihood of postsurgical seizure control for patients with MRI lesional extratemporal epilepsy compared to NLET (72% compared to 41% [9], 53% compared to 17% [10], and 66% compared to 38% [26]).

The absence of a MRI lesion to guide surgery in NLET highlights the importance of scalp EEG and intracranial EEG (iEEG) ictal onset patterns. Previous investigations [19, 27–31] of MRI lesional and NLET have previously focused on iEEG in an attempt to identify specific features related to surgical outcome. In this study, we identified a cohort of patients who underwent resective surgery for NLET and analyzed clinical information, functional imaging, and the electrophysiological features of both scalp and iEEG interictal and ictal patterns, including seizure onset oscillation frequency, spatial distribution, and waveform morphology. 

## 2. Methods

Between 1997 and 2007, 41 consecutive patients with normal MRI underwent chronic iEEG monitoring for presurgical evaluation of medically resistant extratemporal lobe epilepsy. Patients were identified by a retrospective review of the Mayo Clinic electronic record system. Each patient gave informed consent for participation in this Mayo Clinic Institutional Review Board approved study. The demographics of patients at the time of surgery, lobar localization of epilepsy, duration of epilepsy, presence of epilepsy risk factors, seizure semiology from video-EEG (vEEG), scalp and intracranial EEG ictal onset pattern, interictal and ictal SPECT, Subtraction ictal SPECT coregistered to MRI (SISCOM) [32], and International League Against Epilepsy (ILAE) outcome score [33] were abstracted from the Mayo Rochester Epilepsy Surgery Database. 

All patients underwent comprehensive non-invasive evaluation, including wake and sleep interictal EEG with standard-activating procedures, seizure protocol MRI, neuropsychological studies, and long-term vEEG monitoring to record habitual seizures. A standardized seizure protocol MRI was performed with a 1.5-Tesla Signa Scanner (GE Medical Systems, Milwaukee, WI, USA) [34]. This protocol included a spin echo T1-weighted whole-brain volumetric series consisting of 124 contiguous 1.5-mm-thick slices, either coronal or perpendicular acquisition along the long axis of the hippocampal formation. Axial T1-weighted images, coronal and axial T2-weighted and proton density images, and coronal FLAIR images were acquired with a 3-mm slice thickness and a 2-mm interslice gap. Interictal and ictal SPECT and SISCOM [32, 35] were performed in all patients.

Prior to chronic iEEG monitoring each patient was reviewed at a multidisciplinary epilepsy surgery conference attended by epileptologists, neuroradiologists, neuropsychologists, and neurosurgeons. The clinical decision for implantation and spatial distribution of intracranial electrodes during phase II evaluation was generated at the conference. Patients had intracranial electrodes placed according to the hypothesis developed from the noninvasive presurgical evaluation [6, 7, 36, 37]. Ad-Tech subdural grids, strips, and depth electrodes (Ad-Tech Medical Instrument Corp.) were used for all studies. The placement of subdural and depth electrodes was based on findings from the noninvasive studies. 

The subdural grid electrode contacts were 4.0 mm diameter Platinum-Iridium discs with a center-to-center electrode distance of 10 mm. Continuous video-EEG and iEEG were collected using 1 of 2 clinical digital acquisition systems: XLTEK (XLTEK Corp.; 128-channel 16-bit A/D, and 500 Hz sampling frequency) or the NCI (Lamont Medical; 128-channel, 16-bit A/D and 250 Hz sampling frequency). Bipolar and referential montage recordings were reviewed, and in the majority of cases the bipolar montage was used to eliminate common mode artifact. A digital 60-Hz notch filter was used when necessary to eliminate line noise. A neuropathologist provided the pathological diagnosis of resected tissue.

### 2.1. Ictal and Interictal EEG Classification

Scalp and iEEG recordings were visually reviewed while blinded to all clinical and outcome information. Interictal scalp EEG was classified into 4 patterns of interictal epileptiform discharges (IEDs): (1) patients with localized IEDs, (2) patients with lateralized but not localized IEDs, (3) patients with generalized or bilateral IEDs, (4) patients without IEDs. Surgery outcome was analyzed for these groups independently, and between group 1 and combined groups 2, 3, and 4.

Seizure onset times were determined by visually identifying a clear electrographic seizure discharge, and then looking backward in time for the earliest EEG change. The earliest EEG change was labeled as the seizure onset time. Ictal onset pattern was defined within the first 5 seconds of a localized, sustained EEG pattern that was visually distinguished from background activity, and ultimately accompanied by objective or subjective clinical manifestations. Ictal onset patterns were characterized by the seizure discharge oscillation frequency, waveform morphology, and spatial distribution. The seizure onset zone, defined by the electrode(s) with the earliest seizure activity, was classified as frontal, parietal, or occipital. 

Seizure onset frequency was classified within the initial 5 seconds of onset into gamma (>30 Hz), beta (14–29 Hz), alpha (8–13 Hz), theta (5–7 Hz), or delta (<4 Hz) [38]. Regrouping as fast (>13–125 Hz) and slow (<13 Hz) frequencies was done for further statistical analysis because of the small patient numbers [29]. Spatial distribution of EEG seizure onset was classified as either focal onset (involvement of one lobe on scalp EEG or less than five contacts on iEEG), or diffuse, according to the number and the location of electrode contacts showing the earliest involvement in each seizure. Epilepsy surgery outcome was classified using ILAE scoring system [33]. In this study the ILAE class 1 (seizure-free), 2 (seizure free except auras), and 3 (3 or less seizure days per year) were considered favorable, and ILAE 4–6 class outcomes unfavorable (Table 1).

### 2.2. Data Analysis

Postoperative seizure outcome was investigated using Kaplan-Meyer survival analysis [39]. To study potential predictors of favorable resective epilepsy surgery outcome, the following variables were investigated in univariate Cox-proportional hazard models: duration of epilepsy, presence of epilepsy risk factors, seizure semiology from video EEG, scalp and intracranial ictal onset patterns, and spatial distribution, SPECT, and SISCOM. Statistical analyses were performed using JMP (version 7.0, SAS Institute) and *P* ≤ 0.05 was considered statistically significant. 

## 3. Results

**Figure 1:**
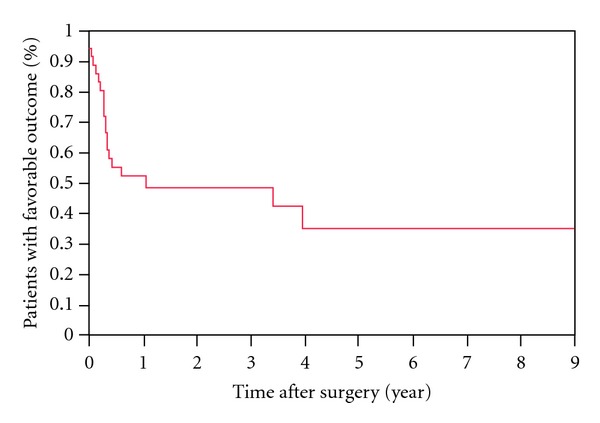
Kaplan-Meier survival curve. A favorable surgical outcome was reported by 49% of patients after 1 year and by 35% after 4 years of followup.

Thirty-six of the 41 patients had adequate information with a minimum of 1-year followup. Twenty-eight (28/36, 78%) had frontal lobe epilepsy, six (6/36, 17%) had parietal lobe epilepsy, and two (2/36, 5%) had occipital lobe epilepsy. The pathology was either nonspecific gliosis (25/36, 70%) or cortical dysplasia (11/36, 30%). Patient characteristics are shown in Table 2. A favorable surgical outcome, ILAE score 1–3, was reported by 49% after 1 year and 35% after 4 years of followup (Figure 1). Antiepileptic medications were successfully tapered and discontinued in only one patient. No association was found between favorable outcome at 1 year and the following variables: seizure semiology (presence of generalized tonic clonic seizures), epilepsy duration, age of onset of seizures, interictal scalp EEG findings, nonspecific MRI abnormalities, SISCOM, pathology, and postoperative EEG epileptogenic abnormalities (Table 3). 

**Figure 2:**
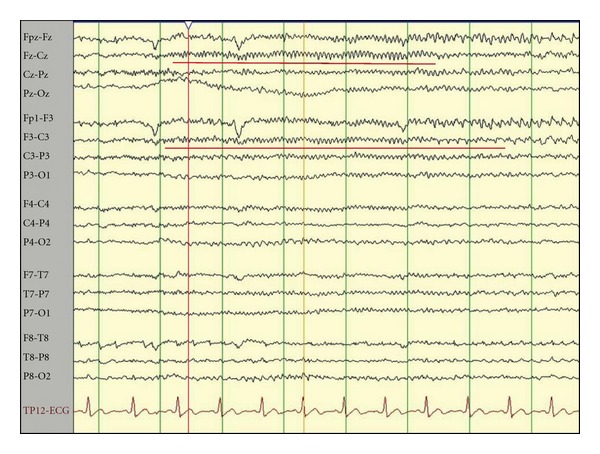
Scalp EEG-recorded extratemporal onset seizure. The bipolar montage-recorded seizure onset (red horizontal line) shows a focal ictal beta (FIB) frequency discharge from the left frontocentral (Fz, F3, Cz) head region.

**Figure 3:**
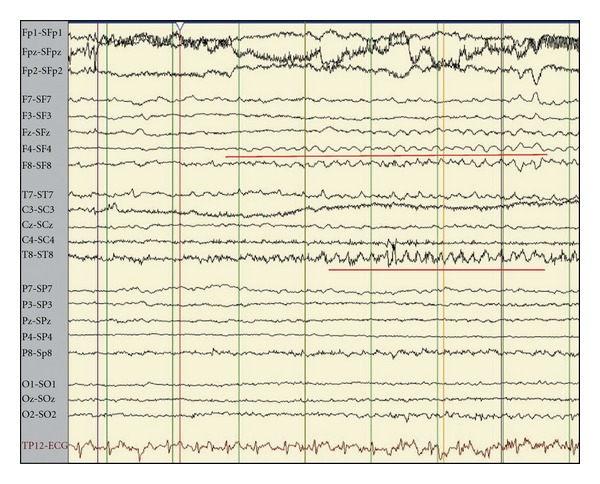
Scalp EEG-recorded extratemporal onset seizure. The Laplacian montage-recorded seizure onset (red horizontal line) shows a theta frequency rhythmic discharge from the right frontotemporal (F4, F8, T8) head region.

**Figure 4:**
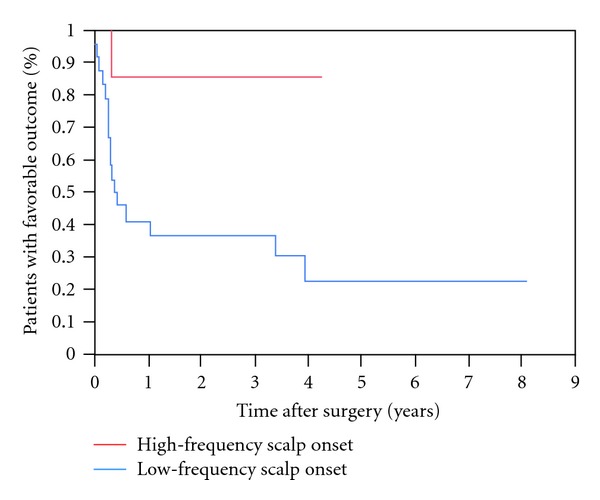
Kaplan-Meier survival curves comparing patients with scalp-recorded focal ictal beta frequency onset (>13 Hz) (red line) and low frequency (≤13 Hz) (blue line).

**Figure 5:**
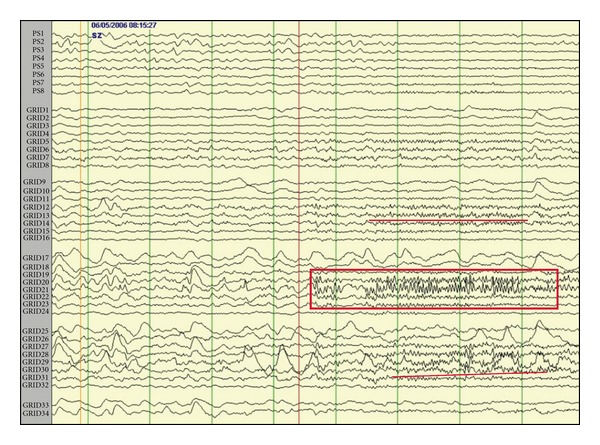
Subdural intracranial EEG-(iEEG) recorded frontal lobe onset seizure. Referential montage iEEG from 6 × 6 (36-contact) subdural grid (contacts 35 and 36 removed because of not recording) and a 1 × 8 (8 contact) subdural strip. Gamma frequency oscillation at seizure onset (red box) primarily involving contacts 21, 22, and 23 with early spread to contacts 12, 13, 28, 29, and 30.

**Figure 6:**
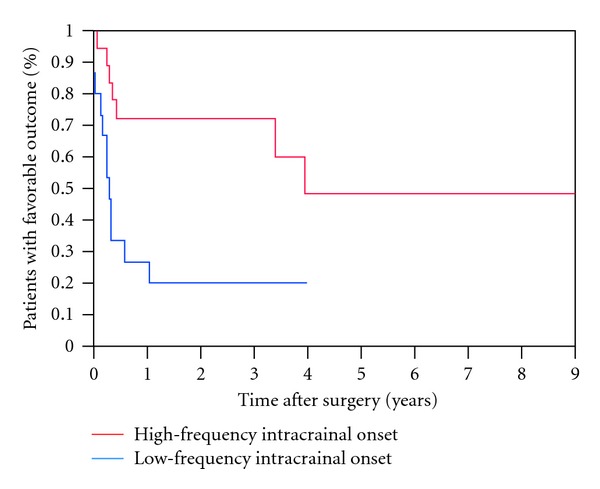
Kaplan-Meier survival curves comparing patients with intracranial EEG recorded fast frequency oscillation at seizure onset (>14 Hz, red line) and low frequency onset (<14 Hz, blue line).

The only presurgical prognostic factors associated with favorable outcome *(P < 0.05)* were scalp and intracranial EEG ictal onset patterns. A focal ictal beta frequency discharge on scalp EEG at seizure onset (*P* = 0.03, Figures 2, 3, and 4) and restriction of seizure onset to one anatomic lobe (*P* < 0.001) were associated with favorable outcome. Similarly, a fast frequency (>13–125 Hz), discharge on iEEG at seizure onset (*P* = 0.02, Figures 5 and 6) and focal seizure onset zone involving 5 or less contiguous intracranial electrodes (*P* = 0.03) were associated with a favorable outcome.

## 4. Discussion

Nonlesional extratemporal lobe epilepsy represents one of the most challenging patient groups seen in clinical practice. Surgical series from multiple institutions have demonstrated the prognostic importance of an intracranial lesion on MRI, and that excellent surgical outcomes are achievable provided that the structural lesion can be completely resected [14, 15, 40–43].

However, the preoperative MRI is normal in approximately 20–30% of patients with medically resistant epilepsy presenting to tertiary epilepsy centers [26, 44]. When the MRI is nonlesional, defining the epileptogenic zone is difficult and epilepsy surgery outcomes are inferior to lesional epilepsy surgery. Here we investigated the clinical, EEG, and functional imaging findings associated with favorable outcome in NLET. The goal was to identify potentially useful diagnostic features for favorable surgical outcome.

We identified electrophysiologic features of scalp and intracranial EEG seizure onset that were associated with favorable surgical outcome. The presence of a focal, fast frequency oscillation at ictal onset was associated with a favorable outcome.

### 4.1. Scalp Interictal EEG

Several studies have reported that the presence of generalized IEDs and/or generalized slowing of the background activity [45] are related to poor outcome in neocortical extratemporal epilepsy surgery, whereas the presence of focal IEDs has been associated with excellent outcome [23, 24]. In contrast, several other studies, including our study, did not find an association between the spatial distribution of IEDs and surgery outcome [6, 7, 17]. 

The variability in previously reported results could be related to the widespread distribution of IEDs in patients with extratemporal epilepsy. A previous study from our center showed that 55% of frontal lobe epilepsy patients had either generalized IED or focal IED in regions other than the epileptogenic zone [46]. It has been established that an epileptic focus in the deep frontal lobe can produce secondarily generalized IEDs with rapid interhemispheric spread [47]. Despite the presence of generalized IEDs, these cases can still benefit from frontal lobe resections [46, 48]. and as many as 65% of patients with resectable lesions and bilaterally synchronous IEDs in preoperative interictal EEG remained seizure-free 5 years after surgery [12]. On the other hand, the absence of IEDs could be related to a small focal region of epileptogenic brain that does not generate detectable IEDs on scalp EEG. Previous studies have shown that approximately 7 cm^2^ of neocortex must be activated to produce IEDs on scalp EEG [49]. From these studies, we could conclude the presence of widespread IEDs, or the absence of IEDS should not preclude patients from consideration for epilepsy surgery.

### 4.2. Scalp Ictal EEG Onset Pattern

The results reported here are in agreement with the limited number of studies in the literature investigating scalp EEG ictal onset pattern and surgery outcome. Talairrach et al. [14] concluded that a fast-frequency ictal discharge was associated with successful epilepsy surgery in a group of patients with predominantly lesional frontal lobe epilepsy. Similarly, we previously reported that a focal beta frequency (>13–25 Hz) discharge at ictal onset, that is, focal ictal beta (Figure 2), was associated with excellent outcome [50] in lesional and nonlesional frontal lobe epilepsy. 

### 4.3. SISCOM

Previous studies that included patients with lesional and NLET patients found an association between outcome and complete resection of a SISCOM abnormality [32]. Here we studied a group of patients with NLET and did not find a definite association between a localizing SPECT/SISCOM and seizure outcome. A possible explanation is the small number of patients, and the bias introduced by the fact that NLET patients with positive SPECT and SISCOM studies are more likely to be considered for invasive monitoring and epilepsy surgery. Additionally, in the current study we did not evaluate the degree of resection of the SISCOM abnormality [32]. 

### 4.4. Intracranial EEG

In our series, a focal fast frequency ictal onset pattern on iEEG (Figure 5) was associated with favorable outcome in NLET epilepsy surgery. Two previous studies have investigated iEEG ictal onset patterns and surgery outcome in patients with NLET [30, 31]. Consistent with the study reported here, the ictal onset patterns consisting of focal low voltage fast or high amplitude beta-frequency spiking was associated with favorable surgical outcome, whereas rhythmic sinusoidal activity or rhythmic spike/sharp waves of slow-frequency were associated with poor outcome [30, 31].

It has been postulated that EEG electrodes showing faster-frequency ictal discharge at seizure onset might be closer to the seizure-onset zone than electrodes recording slower-frequency activity [51]. Indirect support for this hypothesis comes from several studies of mainly temporal lobe epilepsy surgery indicating that low-voltage fast activity at seizure onset was associated with good surgical outcome [31, 52–55]. In contrast, slower-frequency diffuse activity at ictal onset may suggest a propagated electrographic pattern [52, 56].

Human iEEG studies performed as part of presurgical evaluations have primarily focused on a limited dynamic range (~0.1–40 Hz) [57]. Recently, the use of broadband digital EEG recordings has allowed observation of very high-frequency activity (>80 Hz) at the onset of neocortical seizures [4, 51, 53, 55, 58, 59]. Several reports support that high-frequency discharges above 250 Hz are pathological and may be a marker for epileptogenic brain [58–62]. Whether modification of the surgical resection margin to include brain regions generating these very high-frequency interictal and ictal discharges can improve epilepsy surgery outcomes is an active area of research.

## 5. Conclusion

Despite advances in MRI technology, a significant number of patients with medically resistant partial epilepsy have normal MRI. Patients with NLET represent one of the most challenging groups of patients because of the difficulty localizing the epileptogenic zone. Here we report that the presence of a focal, fast-frequency discharge at seizure onset on scalp or intracranial EEG is useful for localizing the epileptogenic zone and associated with favorable surgical outcome in patients with NLET.

## Figures and Tables

**Table 1:** ILAE classification of outcome.

(1) Completely seizure free; no auras	
(2) Only auras; no other seizures	Favorable outcome
(3) One-to-three seizure days per year; ±auras	

(4) Four seizure days per year to 50% reduction of	
baseline seizure days; ±auras	
(5) Less than 50% reduction of baseline seizure days to	Unfavorable outcome
100% increase of baseline seizure days; ±auras	
(6) More than 100% increase of baseline seizure days;	
±auras	

**Table 2:** Clinical characteristics of patients.

Clinical characteristics	*N* (%)	Mean	Range
	36 patients		
Age at surgery, years		27	8–71
Age at onset		7.4	1–19
Duration		19.5	2–52
Female	14 (38%)		
Location:			
frontal	28 (78%)		
parietal	6 (17%)	
occipital	2 (5%)	
Presence of risk factor	14 (38%)		
Presence of GTC seizure	14 (38%)		
Pathology:			
gliosis	25 (70%)		
MCD	11 (30%)		
Scalp EEG:			
Group 1	12 (33%)		
Group 2	8 (22%)	
Group 3	9 (25%)	
Group 4	7 (15%)	
Ictal scalp EEG patterns:			
focal Ictal Beta	6/30 (20%)	
other	24/30 (80%)	
iEEG onset patterns			
Fast frequency (>14 Hz)	17/31 (55%)		
Other	14/31 (45%)		
Localized SPECT	13 (36%)		
Localized SISCOM	22 (61%)		

GTC: generalized tonic clonic seizure.

MCD: malformation of cortical development.

IED: interictal epileptiform discharge.

Group 1: localized IED; Group 2: lateralized but not localized IED; Group 3: generalized or bilateral IED; Group 4: no IED.

**Table 3:** Analysis of surgery outcome at 1-year followup.

Variables of interest	Favorable versus	*P* value
	unfavorable outcome	
Age of Onset:	40% versus 50%	0.54
<10 yrs		
>10 yrs		
Scalp interictal EEG:
Group 1 (localized IED)	50% versus 40%	0.25
Group 2, 3, and 4 (other)		
Seizure semiology:
history of GTC	50% versus 49%	0.87
no history of GTC		
Duration of epilepsy:
>15 years	60% versus 40%	0.19
<15 years		
Pathology:
cortical dysplasia	50% versus 49%	0.51
gliosis		
Nonspecific MRI abnormality:
absent	50% versus 48%	0.9
present		
SISCOM perfusion abnormality:
localized	61% versus 45%	0.88
nonlocalized		
Postoperative scalp EEG	55% versus 50%	0.32
IED present versus negative		

IED: interictal epileptiform discharge

Group 1: localized IED; Group 2: lateralized but not localized IED; Group 3: generalized or bilateral IED; Group 4: no IED.
